# The role of KIBRA in reconstructive episodic memory

**DOI:** 10.1186/s10020-018-0007-8

**Published:** 2018-03-15

**Authors:** Armin Zlomuzica, Friederike Preusser, Susanna Roberts, Marcella L. Woud, Kathryn J. Lester, Ekrem Dere, Thalia C. Eley, Jürgen Margraf

**Affiliations:** 1 Mental Health Research and Treatment Center, Ruhr-Universität Bochum, 150, 44780 Bochum, Germany; 2 Institute of Psychiatry, Psychology and Neuroscience, MRC Social, Genetic and Developmental Psychiatry Centre, King’s College London, London, UK; 3 School of Psychology, University of Sussex, Brighton, UK; 4 Teaching and Research Unit. Life Sciences (UFR927), University Pierre and Marie Curie, Paris, France; 5 Clinical Neuroscience, Max Planck Institute of Experimental Medicine, Göttingen, Germany

**Keywords:** KIBRA, WWC1 gene, Episodic memory, False memories, Alzheimer’s disease

## Abstract

**Background:**

In order to retrieve episodic past events, the missing information needs to be reconstructed using information stored in semantic memory. Failures in these reconstructive processes are expressed as false memories. KIBRA single nucleotide polymorphism (rs17070145) has been linked to episodic memory performance as well as an increased risk of Alzheimer’s disease and post-traumatic stress disorder (PTSD).

**Methods:**

Here, the role of KIBRA rs17070145 polymorphism (male and female CC vs. CT/TT carriers) in reconstructive episodic memory in the Deese-Roediger-McDermott (DRM) paradigm was investigated in *N* = 219 healthy individuals.

**Results:**

Female participants outperformed males in the free recall condition. Furthermore, a trend towards a *gender x genotype interaction* was found for false recognition rates. Female CT/TT carriers exhibited a lower proportion of false recognition rates for associated critical lures as compared to male CT/TT. Additionally, an association between KIBRA rs17070145 genotype, familiarity and recollection based recognition performance was found. In trials with correct recognition of listed items CT/TT carriers showed more “remember”, but fewer “know” responses as compared to CC carriers.

**Discussion and conclusion:**

Our findings suggest that the T-allele of KIBRA rs17070145 supports recollection based episodic memory retrieval and contributes to memory accuracy in a gender dependent manner. Findings are discussed in the context of the specific contribution of KIBRA related SNPs to reconstructive episodic memory and its implications for cognitive and emotional symptoms in dementia and PTSD.

## Background

Episodic memory refers to the ability to recollect personal experiences and specific events in terms of contextual details, specific perceptions, emotions, and thoughts (Tulving, 2002). Genetic variability seems to play a prominent role in the inter-individual variation in episodic memory performance (Volk et al., 2006). However, the relevant genes, gene clusters and their molecular pathways remain to be determined.

Recently, a genome-wide association study identified KIBRA as a potential candidate gene that is associated with the encoding and retrieval of episodic memories (Papassotiropoulos & de Quervain, 2011). KIBRA is abundantly expressed in brain regions such as the prefrontal cortex and hippocampus that are at the core of episodic memory formation and retrieval (Papassotiropoulos et al., 2006). In the hippocampus, the KIBRA gene is expressed in neurons. The KIBRA protein interacts with synaptopodin (Duning et al., 2008) and PKCζ (Büther et al., 2004), which are both involved in synaptic plasticity. An important role of KIBRA in development of brain architecture (Yoshihama et al., 2009; Johannsen et al., 2008) has also been confirmed. Genetic deletion of KIBRA in mice leads to impairments in hippocampal long-term potentiation as well as compromised memory performance (Makuch et al., 2011). At the behavioral level, a considerable number of studies confirmed an association between a single nucleotide polymorphism (SNP) of the KIBRA gene (rs17070145) and episodic memory performance, with T-allele carriers (CT/TT) of the SNP showing a superior performance relative to non-carriers (CC) (summarized in Milnik et al., 2012). However, findings regarding KIBRA and memory functions are inconclusive and depend on whether young or older adults are being examined and whether participants are healthy or suffer from neurological or neurodegenerative diseases (Nacmias et al., 2008; Need et al., 2008; Boraxbekk et al., 2015; Rodriguez-Rodriguez et al., 2009; Schaper et al., 2008); Vassos et al., 2010 summarized in Schwab et al., 2014. Imaging studies suggest that the genetic variation in KIBRA rs17070145 is also related to differences in patterns of brain activation during episodic retrieval, particularly in the hippocampal/medial temporal lobe region (Papassotiropoulos et al., 2006; Kauppi et al., 2011).

Since our capacity to recollect complex personal events is limited, episodic memories often contain only a selection or fragments of the original event information. With regard to retrieval, such fragmentary information has to be complemented with semantic information in order to reconstruct the original event as precise as possible (Pause et al., 2013; Loftus, 2005; Breeden et al., 2016). Errors during the reconstruction process of past events are expressed as false memories, i.e., subjects often tend to recall false information or recognize items incorrectly simply because they are semantically or visually related to correct information or items that were actually presented.

The DRM task was developed as in attempt to design a simple and fast task to induce and measure false memories under laboratory conditions (Deese, 1959; Roediger & McDermott, 1995). The DRM experimental procedure (see Pardilla-Delgado, 2017) can be easily applied (without further adaptations necessary) to children, adults, aged individuals, as well as to amnesic, neurological and psychiatric patients. To date, the DRM paradigm is considered *as a* gold standard for the investigation of psychological and biological factors underlying reconstructive processes (Gallo, 2010). In the DRM task, participants are first instructed to memorize lists of semantically related words (e.g., “dark”, “night”, etc.). Each of the words presented during the learning phase is highly associative of a word belonging to the gist or theme of the respective word list (referred to as the critical lure word: “black”). The critical lure representing the gist or theme of the lists of semantically related words, however, is not being presented. During the subsequent test phase, subjects often tend to recall and/or recognize both the unpresented critical lure word (false memories) and words presented during the initial learning phase (accurate memories). The reliability of the DRM task in creating false memories is well documented and reflected by its predominant use in false memory research.

Determining individual differences with respect to susceptibility to produce false memories in the DRM paradigm has a long tradition in the field (Deese, 1959; Roediger & McDermott, 1995). A number of decisive factors, including increased dissociative and delusional tendency (Laws & Bhatt, 2005; Winograd et al., 1998), high schizotypy (Saunders et al., 2012), and specific autobiographic memory retrieval style (Dewhurst et al., 2018), have been identified that predict high false recognition rates in healthy subjects. Contrarily, the contribution of genetic factors to individual differences in reconstructive episodic memories has been largely neglected (but see Zhu et al., 2013). Recently, Zhu et al., 2013 demonstrated that the 5-Hydroxytryptamine Receptor 2 (HTR2A) gene is significantly associated with the capacity to retrieve true, but not false memories during recognition in the DRM task.

So far research on the association between KIBRA rs17070145 and episodic memory has neglected the reconstructive nature of episodic memory functions. Both, the storage and retrieval of veridical and false episodic memories (as studied in the DRM paradigm) are supposed to be subserved by distinct brain regions, i.e. anterior and posterior regions of the medial temporal lobe, dentate gyrus and CA subregions of the hippocampus (Schacter et al., 1996; Schacter & Slotnick, 2004; Ramirez et al., 2013). Since KIBRA is abundantly expressed in the CA1 region of the hippocampus as well as the dentate gyrus (Papassotiropoulos et al., 2006; Yoshihama et al., 2009; Johannsen et al., 2008), suggesting a possible involvement of KIBRA in processes related to reconstructive episodic memory, we asked whether KIBRA related genotype effects exist for reconstructive episodic memory and whether these can be observed during recall and recognition memory performance in the DRM task.

The precise role of the hippocampus and its adjacent areas in recollection and familiarity-based recognition memory is still a matter of debate. Results from numerous neuropsychological, neuroimaging and neurophysiological studies implicate that the hippocampus and posterior parahippocampal cortex selectively support recollection-based recognition, whereas other regions (e.g. the rhinal cortex) mediate familiarity-based recognition (Ranganath et al., 2004; Suchan et al., 2008). Notably, there is preliminary evidence from imaging studies that KIBRA is associated with structural differences in areas which are selectively involved in recollection and familiarity-based recognition memory (Palombo et al., 2013). To explore the possibility that qualitative aspects of recognition performance might be differentially affected by KIBRA polymorphism, the remember/know judgment procedure was employed in our study.

Considerable evidence suggests that episodic memory performance differs between genders, with women showing superior retrieval in dependence of the learning material, i.e. verbal, spatial or autobiographical information (Andreano & Cahill, 2009; Herlitz et al., 1997). Furthermore, the association between KIBRA rs17070145 polymorphism and cognitive functions is more pronounced in healthy female subjects as compared to males or mixed samples (Wersching et al., 2011). We thus included gender as an additional important factor to determine the association between KIBRA and reconstructive episodic memory.

## Methods

### Participants

A total of *N* = 219 healthy students with no history of psychiatric disease and/or current psychoactive medication were tested. Twelve participants were excluded from analysis because they could not be genotyped (*n* = 5) or had incomplete test data (*n* = 7). Each participant was instructed to refrain from eating food and drinking beverages (except for water) 1 h prior to the experiment. Subjects received either financial allowance (10euro/h) or course credits for participation. All participants provided written informed consent. The study was approved by the local ethics committee of the University of Bochum and was carried out in accordance with the principles outlined by the Declaration of Helsinki.

### Experimental procedure

Participants were tested in the DRM paradigm according to a slightly modified procedure by Roediger & McDermott, 1995. Briefly, all subjects were instructed to memorize word lists for a subsequent memory test. The learning material comprised 8 word lists which were derived from Stadler et al., 1999 word list inventory and translated to German. Each word list consisted of 15 words which were semantically related to a specific theme word, which was not presented during the learning phase itself (e.g. “cold” was a theme word and was not presented, but instead its highly associative words “hot”, “snow”, “warm” etc. were presented).

Words of each list were presented both as auditory (via earphones by a pre-recorded female voice) and visual stimuli on the computer screen. Words of a list were presented with an inter-word delay of 750 ms, whereas word lists as a whole were presented with a delay of 10 s. The order of word presentation was chosen according to the associative strength with the theme word (from the associatively strongest word to the weakest one). After the learning phase, each participant completed an unrelated distraction task for approximately 15 min to prevent rehearsal. Subsequently, each participant was asked to recall as many words as possible from the initial learning phase and to write down these words on a sheet of paper. Participants were granted 4 min to recall and write down the words. After another distraction task, participants completed the recognition test. During the recognition test, words that had been presented at serial positions 1, 5 and 10 of each word list during the initial learning phase (“listed items”) as well as unrelated distractor words (“distractor items”, i.e. words not presented during encoding and unrelated to listed items) and semantically-associated theme words (“critical lures”, i.e. words not presented during encoding but highly related to listed items) were presented individually on a sheet of paper. Participants were asked to rate each word as old or new (i.e. according as to whether the word had been presented during the learning phase or not) as well as to categorize the words judged as “old” according to the Remember/Know/Guess procedure (Seamon et al., 2002).

### Questionnaires

In order to control for the impact of depressive symptoms, trait anxiety and stress sensitivity on the retrieval of accurate and false memories, each participant completed specifically selected items from the Depression Anxiety Stress Scales (DASS; Lovibond & Lovibond, 1995) prior to the encoding phase.

### Genotyping

DNA samples were collected using OG-100 Oragene saliva collection kits (DNA Genotek, Ontario, Canada). DNA extraction and genotyping were performed using established procedures according to the manufacturers protocol. The KIBRA rs17070145 polymorphism was genotyped by LGC Genomics (Hoddesdon, UK) using KASP technology with validated arrays. Five participants could not be genotyped, giving a genotyping success rate of 97.7%.

### Statistical procedures

Statistical procedures were conducted with the software package SPSS statistics Version 22 (IBM; Armonk, NY, USA: IBM Corp.). All analyses were performed using the dominant model of inheritance (CC vs. CT/TT). With respect to free recall, the proportion of listed items and critical lure items were entered as within-subjects factors while Genotype (CC vs. CT/TT) and Gender were entered as between-subjects factor in a mixed-design ANOVA. Measures of recognition memory (hit rates (=listed items classified as “old”; false memory rates (=critical lures classified as “old”; and false alarm rates (=distractor items classified as “old”) were corrected prior to analyses according to the procedure by Snodgrass & Corwin, 1988. Genotype and gender differences in these recognition memory scores were assessed using mixed ANOVAs and a series of univariate analyses. Remember/Know/Guess Judgments were analyzed by a series of 2 (Genotype) × 2 (Gender) × 3 (Item-type; critical lures, distractors, listed items) mixed ANOVAs. Bonferroni-correction for multiple testing was applied where indicated and simple effects analyses were conducted following a significant interaction or main effect. Where appropriate, degrees of freedom were corrected by Greenhouse-Geiser estimates of sphericity. *P*-values < 0.05 were considered to be significant.

## Results

For the KIBRA polymorphism, 97 subjects were homozygous for the C allele, 26 for the T allele, while 84 subjects were heterozygous for the C/T alleles. The distribution of allele frequencies (C = 67.15%, T = 32.85%) and the hereby observed genotypes were in Hardy-Weinberg equilibrium, *P* = 0.2486.

As displayed in Table 1, CC and CT/TT carriers were comparable with respect to age, gender distribution and their scores on any of the subscales of the DASS, all P ≥ 0.291.

**Table 1:** Demographic characteristics of the different genotypes

Variable	CC (n = 97)	CT/TT (n = 110)	*P -* value
	M (SD)	M (SD)	
Age (years)	24.47 (4.87)	25.34 (6.59)	0.291
Gender % female)	53.6%	56.4%	0.398
DASS			
Depression	2.38 (2.53)	2.82 (3.45)	0.306
Anxiety	2.76 (2.97)	2.51 (2.52)	0.507
Stress	6.07 (3.99)	6.46 (4.43)	0.507

*DASS* Depression Anxiety Stress Scales

### Free recall condition

As indicated by a significant main effect for item-type, F(1, 203) = 131.287, *P* < 0.001, subjects recalled a greater proportion of listed items [0.34 ± 0.01 (mean ± SE)] than critical lures (0.15 ± 0.01). Furthermore, we found an item-type x gender interaction, F(1, 203) = 5.033, *P* = 0.026, with female participants (0.36 ± 0.01) recalling a greater proportion of listed items than males (0.31 ± 0.01), *P* = 0.003.

No other main or interaction effects attained statistical significance, all P ≥ 0.215. With respect to the total number of words recalled, females (M = 46.76, SD = 1.31) again outperformed males (M = 41.33, SD = 1.45), F(1, 203) = 7.780, *P* = 0.006, while no effects for genotype were evident (main effect and interaction, all P ≥ 0.243). In addition, genotypes and genders were comparable in the number of distractors being recalled (main or interaction effects, all P ≥ 0.05).

### Recognition memory

**Fig. 1:**
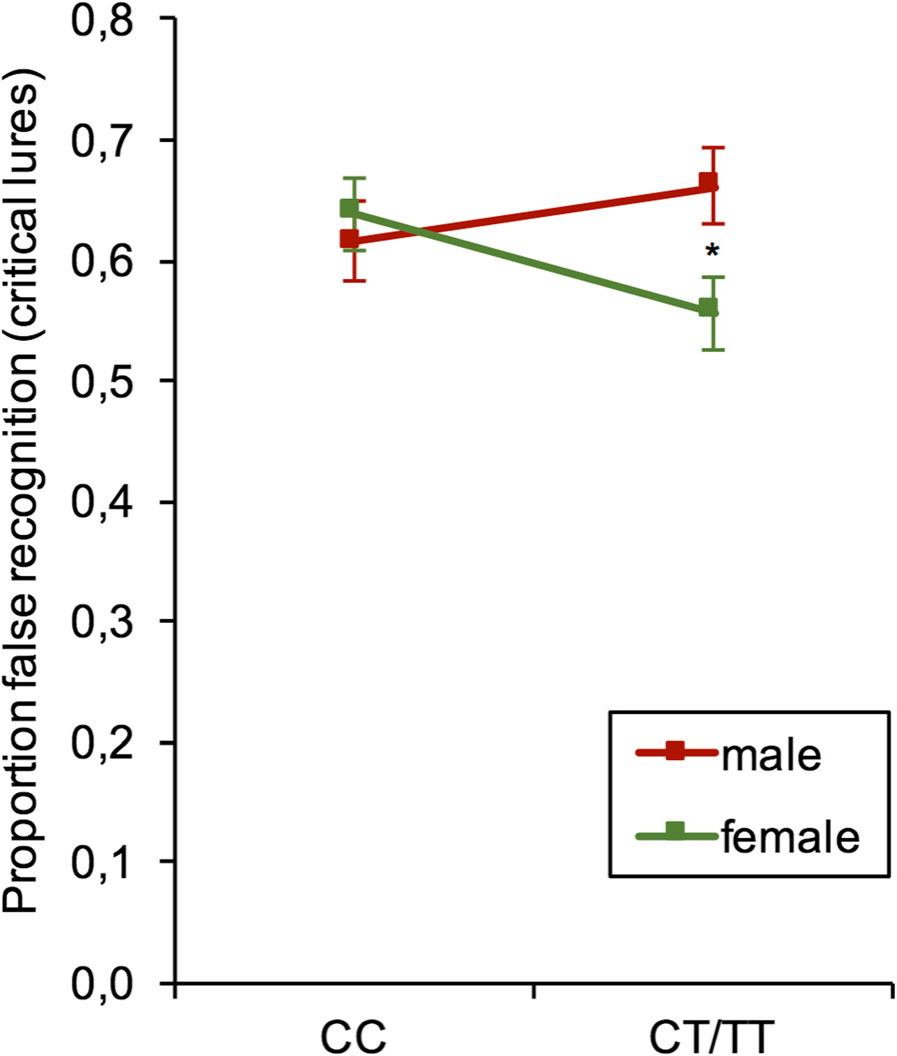
False recognition rates for critical lures. Female but not male CT/TT carriers show a lower proportion of false recognition rates for critical lures as compared to CC carriers. After controlling for repeated testing, the gender x genotype interaction did not remain significant. Data expressed as means ± 1 SEM. * *P* < 0.05

Discriminability scores for hit rates, false memory rates, and false alarm rates in male and female CC and CT/TT carriers are summarized in Table 2. A mixed ANOVA with type of recognition (hit rates, false memories, false alarms) as within-subjects factor as well as genotype and gender as between-subjects factor revealed a significant main effect for type of recognition, F (1.553, 315.258) = 1156.324, *P* < .001 (Greenhouse-Geiser: ε = 0.776), and a significant gender x genotype interaction, F (1, 203) = 4.050, *P* = 0.045, as well as a trend towards a three-way interaction, F(1.553, 315.258) = 2.409, *P* = 0.105 (Greenhouse-Geiser: ε = 0.776). Interestingly, a series of univariate analyses showed that carriers of the T-allele did not differ from CC homozygotes in their hit rates, F (1, 203) = 0.152, *P* = 0.697, and false alarm rates, F (1, 203) = 0.004, *P* = 0.948), with these patterns not being subjected to gender differences (genotype x gender interaction, all P ≥ 0.328). However, the interaction between gender and genotype emerged for false memory rates, F (1,203) = 4.140, *P* = 0.043, while the main effects themselves were non-significant, all P ≥ 0.190. As shown in Fig. 1, there was a modulation of gender-specific effects by rs17070145 genotype, with females being less prone to false memories than males within the group of T-allele carriers (*P* = 0.016). Furthermore, there was a tendency for female carriers of the T-allele to have a lower proportion of false memories than their counterparts of the CC group (*P* = 0.052). After correcting for repeated testing of the different recognition indices (hit rates, false memories, and false alarms), the gender x genotype interaction for false memory rates did not remain significant (P_corr_ = .129).

**Table 2:** Hit rates, false memory rates and false alarm rates

		Hits	False memories	False alarms
		M ± SE	M ± SE	M ± SE
CC	Male	.82 ± .02	.62 ± .04	.13 ± .01
	Female	.83 ± .01	.64 ± .03	.13 ± .02
	Total	.82 ± .01	.63 ± .02	.13 ± .01
CT/TT	Male	.83 ± .01	.66 ± .03	.14 ± .02
	Female	.83 ± .01	.56 ± .03	.11 ± .01
	Total	.83 ± .01	.60 ± .02	.12 ± .01
Total	Male	.83 ± .01	.64 ± .02	.13 ± .01
	Female	.83 ± .01	.60 ± .02	.12 ± .01

### Remember / know / guess judgments

#### Listed items

**Fig. 2:**
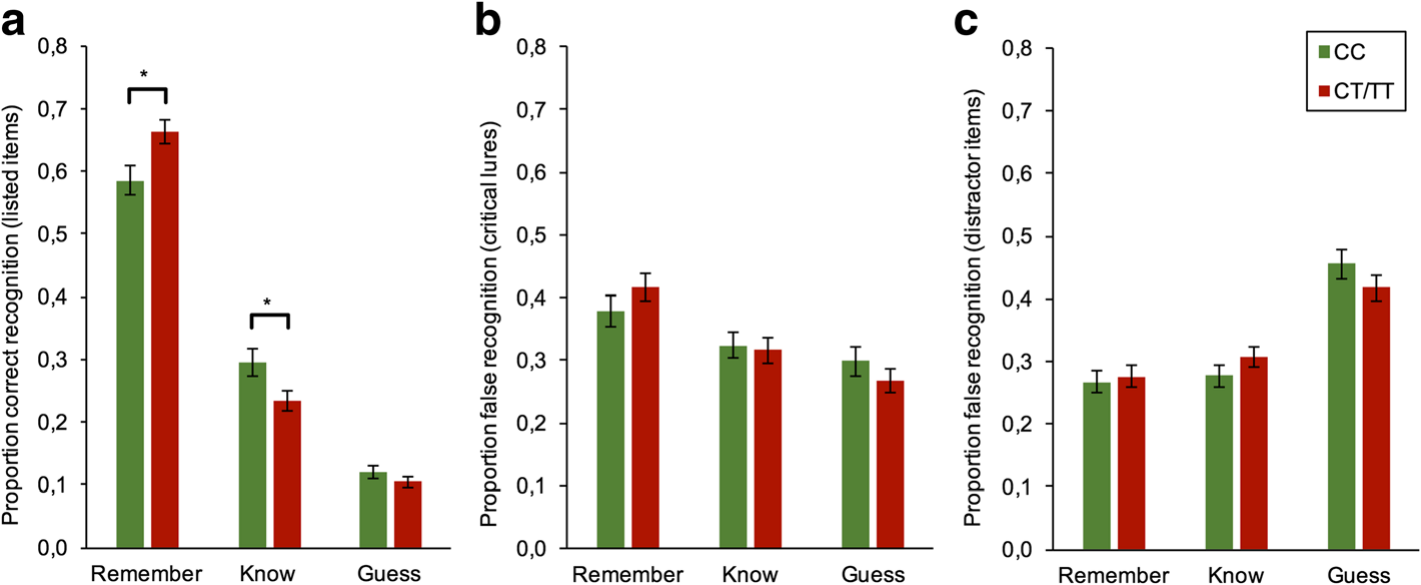
Proportion of Remember / Know / Guess Judgments out of all ‘old’ responses to listed items, critical lures, and distractors in the recognition test. **a**) Remember/know/guess responses during correct recognition trials (listed items). **b**) Remember/know/guess responses during false recognition trials (critical lures). **c**) Remember/know/guess responses during false alarm trials (distractors). Data expressed as means ± SEM. * *P* < 0.05

A significant main effect for response-type, F(1.301, 264.149, = 300.352, *P* < 0.001 (Greenhouse-Geiser: ε = 0.651), and a significant response-type x genotype interaction, F (1.301, 264.149) = 4.891, *P* = 0.019; Greenhouse-Geiser: ε = 0.651) were found. As displayed in Fig. 2a, carriers of the T-allele exhibited a significantly greater proportion of “remember” judgments as compared to CC carriers (P = 0.016), while the opposite was true for “know” judgments (*P* = 0.037). In addition, gender differences (interaction response-type x gender, F (1.301, 246.149) = 3.927, *P* = 0.038; Greenhouse-Geiser: ε = 0.651) were observed for the readout response types. Analysis of simple effects revealed that females had a significantly lower proportion of know responses (*P* = 0.023) and a tendency towards more remember responses (*P* = 0.061) than males.

### Critical lures

As indicated by a significant main effect for response-type F(1.925, 390.749) = 9.789, P < 0.001 (Greenhouse Geiser: ε = 0.962), critical lures were more frequently judged to be remembered than either guessed or known (Fig. 2b). No other effects attained statistical significance, all P ≥ 0.162.

### Distractor items

Only the main effect for response-type, F(1.780, 361.408) = 29.982, P < 0.001 (Greenhouse-Geiser: ε = 0.890), attained statistical significance (all other effects, P ≥ 0.05), with distractors being most frequently subjected to guess judgments (Fig. 2c).

## Discussion

In the present study, we examined the contribution of KIBRA polymorphism in the reconstruction of past episodes as measured by the DRM paradigm. In the free recall condition, we found that female participants outperformed males in terms of total number of items correctly retrieved. This effect is consistent with previous findings on gender differences in episodic memory performance by showing that women show superior performance relative to men, especially on verbal memory tasks (Herlitz & Rehnman, 2008). The explanation for the observed sex differences in episodic memory is still a matter of debate (Herlitz & Rehnman, 2008). Interestingly, accuracy in the correct recognition condition of listed items was not significantly different between males and females or CC and CT/TT carriers of the KIBRA rs17070145 polymorphism. In contrast, we found a significant *gender x genotype interaction* for false recognition rates of critical lures. In particular, female CT/TT carriers exhibited a lower proportion of false recognition rates as compared to male CT/TT carriers and a tendency to be less prone to false recognition of critical lures than female CC carriers. This effect however, did not remain significant after correcting for repeated testing. Thus, our preliminary results tentatively support previous findings on the association between KIBRA polymorphism, gender and cognitive functions (Wersching et al., 2011), but (perhaps due to relatively small sample sizes) failed to reach statistical significance. Results regarding the principal role of KIBRA polymorphism on memory functions are rather inconclusive (summarized in Schwab et al., 2014). In healthy subjects, the T-allele of the KIBRA rs17070145 seem to be associated with significantly better (Schaper et al., 2008; Vassos et al., 2010; Kauppi et al., 2011; Almeida et al., 2008; Preuschhof et al., 2010), slightly better (Witte et al., 2016) or unchanged (Need et al., 2008) declarative memory performance relative to non-carriers of the T-allele. These inconsistencies might be due to methodological differences, i.e. the use of different tasks for the measurement of episodic memory functions. Similar to our results, Wersching et al., 2011 used a neuropsychological test battery and failed to find main effects of the KIBRA rs17070145 genotype on immediate and delayed memory performance. Instead, a significant interaction between gender and rs17070145 genotype was observed for working memory performance (for example in the Digit span test). Furthermore, the same study also reports that gender determines the effect of rs17070145 on executive functioning. Thus, it is possible that our findings regarding false recognition performance are related to an interaction between gender and rs17070145 genotype that modulates working memory and executive functions. Interestingly, working memory capacity seems to be closely related to the susceptibility to false memories (Leding, 2012) and false recognition rates can be significantly reduced by improving executive control functions (McCabe & Smith, 2002). Similarly, Zhang et al., 2009 proposed that the KIBRA rs17070145 T-allele could differentially modulate hippocampal functions such as long-term memory and those functions related to the prefrontal cortex (i.e, working memory capacity). Thus, the putative explanation behind the interactive effects of KIBRA genotype and gender on false recognition performance might be the differential effect of KIBRA in processes related to working memory, executive functions and long-term memory, all of which contribute to differences in the susceptibility to produce false memories. From the methodological perspective, our results thus argue for considerations of gender (Wersching et al., 2011) and task specific demands (Zhang et al., 2009) as important variables in the interpretation of results related to the association of KIBRA and complex cognitive functions such as reconstructive episodic memory.

Another important finding of this study was the association of KIBRA and confidence ratings during recognition memory. While there were no genotype differences in overall recognition performance, the T-allele of the KIBRA rs17070145 polymorphism was associated with differences in qualitative aspects of recognition memory. In particular, in trials with correct recognition of listed items, participants showed significantly more “remember” responses as compared to “know” and “guess” responses. Likewise, “know” responses were significantly higher than “guess” responses during the correct recognition of listed items. Most importantly however, a significant response-type x genotype interaction was evident, indicating that, CT/TT carriers showed more “remember”, but fewer “know” responses as compared to CC carriers during the correct recognition of listed items. One explanation for these findings might be that the T-allele of the KIBRA rs17070145 polymorphism supports recollection-based episodic memory retrieval. According to dual process models of recognition memory, recollection refers to the conscious retrieval of items plus the contextual details encountered during the encoding phase. In contrast, the mere knowing that the item was on the list without remembering the contextual details refers to familiarity-based retrieval processes. Tulving, 1985 proposed that the semantic and episodic memory systems are operating on these two retrieval processes to a different degree. Furthermore, the identified brain structures subserving these different recognition memory processes do not necessarily overlap (Eldridge et al., 2000; Aggleton et al., 2005; Yonelinas et al., 2002). Studies using the remember/know procedure have shown that an intact hippocampus is most probably required for recollection (Aggleton et al., 2005) whereas hippocampal recruitment during familiarity-based retrieval is not obligatory (Ranganath et al., 2004; Suchan et al., 2008). KIBRA is abundantly expressed in the hippocampus. Female T-allele carriers show larger hippocampal volumes relative to non-T-allele carriers (Palombo et al., 2013), an effect which was recently replicated in older healthy adults (Witte et al., 2016). Such structural differences observed in T-carriers and non-carriers of KIBRA rs17070145 might represent an underlying mechanism for the herein observed differences in retrieval characteristics. Indeed, recollection memory can be predicted on the basis of hippocampal longitudinal volume ratios (Poppenk & Moscovitch, 2011). Of course, this conclusion is rather speculative, considering the limited data at hand, especially since we did not perform structural functional magnetic resonance imaging to support this conclusion.

Another compelling but speculative explanation of the behavioral effects observed in the present study could be a genotype-related difference in brain activation patterns. Using a face-profession paired associative learning task (which is intended to measure episodic memory), Papassotiropoulos et al., 2006 found a significantly higher brain activation in the hippocampus and parahippocampal gyrus of CC homozygotes relative to T-carriers during the retrieval but not encoding phase. In a similar task applied to a sample of elderly non-demented subjects, Kauppi et al., 2011 also found differences in hippocampal activation between CC homozygotes and T-allele carriers during retrieval. However, in contrast to the findings obtained by Papassotiropoulos et al., 2006, both a lower activation of medial temporal lobe regions as well as a slower response time was reported in CC homozygous (Kauppi et al., 2011). Hence, it can be concluded that KIBRA rs17070145 genotype-related differences in brain activation patterns exist which do not necessarily lead to equivalent genotype-related differences in episodic memory performance (Papassotiropoulos et al., 2006; Kauppi et al., 2011). Diminished hippocampal functioning in CC homozygous carriers (Papassotiropoulos et al., 2006; Kauppi et al., 2011) corroborate our results, implicating a beneficial effect of KIBRA T-allele in episodic memory mainly due to a qualitatively different retrieval process (i.e. recollection based retrieval) and thus more efficient hippocampal recruitment during recognition performance. Thus, it would be valuable to implement remember/know judgements in future imaging studies to get more insight into the functional link between KIBRA, hippocampal structure and functionality and episodic memory processing.

The following limitations of this study need to be considered. First, the sample size was relatively small for genetic association studies and the herewith associated limited statistical power might explain why we only found a trend towards a gender x genotype interaction for false recognition rates. Interestingly, studies with much smaller sample sizes report similar trends towards an effect (Witte et al., 2016) or even significant effects (Nacmias et al., 2008; Schaper et al., 2008) of KIBRA polymorphism on cognitive functions. In contrast to previous studies, our DRM task is relatively difficult to implement, laborious and more time-consuming. When taking the latter into account, our sample size is substantially larger relative to other genetic association studies employing the DRM procedure (see (Montag et al., 2014). Nevertheless, future studies with larger samples would be beneficial to derive definite conclusions regarding the role of gender and KIBRA genotype on reconstructive memory in the DRM task.

Furthermore, it is conceivable that other genetic factors might play a role in the relationship between KIBRA polymorphism, gender and reconstructive memory. For example, it was repeatedly shown that another gene, CLSTN2 (calsyntenin 2) interacts with KIBRA to modulate episodic memory performance in healthy individuals (Papassotiropoulos et al., 2006; Preuschhof et al., 2010) and depressed elderly subjects (Pantzar et al., 2014). Similar to KIBRA, CLSTN2 is expressed in memory-related brain regions such as the hippocampus (Papassotiropoulos et al., 2006; Hintsch et al., 2002), suggesting a possible involvement in processes related to episodic memory storage and retrieval. Also, we did not employ any structural and/or functional neuroimaging measures, thus any conclusion about underlying neuronal mechanisms mediating the herein observed behavioral effect remain speculative. Such additional measures, however, would be helpful, especially with regard to possible changes in medial temporal lobe/hippocampal activations which go along with our finding of genotype-related differences in familiarity and recollection-based retrieval. Given that the participants in our study were healthy young students the clinical implication of our findings and any extrapolation of the findings are not possible. Nevertheless, there are some important clinical implications that can be derived from this study. It has been shown that non-carriers of the KIBRA rs17070145 T-allele exhibit an increased risk of late-onset Alzheimer’s disease (Corneveaux et al., 2010) which might be related to lower glucose metabolism in brain regions involved in the processing of episodic memories. Similarly, a possible association between KIBRA and the risk of developing strong traumatic memories in survivors of mass conflict (Wilker et al., 2013) has been demonstrated by array-based SNP genotyping. Individuals suffering from PTSD and Alzheimer’s disease show distinct neuropsychological profiles with specific alterations in different aspects of episodic memory functioning. Thus, it could be predicted that the KIBRA (rs17070145) T-allele might have a protective role in both Alzheimer’s disease and PTSD.

## Conclusion

The present study extends previous findings on the possible role of KIBRA on cognitive functions. We add new data showing a trend towards an interactive effect of KIBRA rs17070145 genotype and gender on reconstructive episodic memory, in particular regarding the susceptibility to produce false memories in healthy young adults. Furthermore, we demonstrated an association of KIBRA rs17070145 polymorphism and differences in qualitative aspects of recognition memory, suggesting a beneficial role of the T-allele in supporting recollection-based retrieval processes. We conclude that examining the contribution of genetic variations in KIBRA related SNPs to such systematic alterations in episodic memory functioning might be helpful to counteract the occurrence of cognitive and emotional symptoms in dementia and PTSD (Schneider et al., 2010) and lead to the development of novel therapeutic interventions.
